# McCune-Albright syndrome: a case of an adult with fibrous dysplasia, severe cardiopulmonary complications, acromegaly, and chronic myeloid leukemia

**DOI:** 10.1093/jbmrpl/ziaf090

**Published:** 2025-05-18

**Authors:** Amanda Ji, Anna McLean, Ashim Sinha

**Affiliations:** Department of Diabetes and Endocrinology, Cairns Hospital, Cairns, QLD 4870, Australia; Department of Endocrinology, Mater Hospital Brisbane, Brisbane, QLD 4101, Australia; Department of Diabetes and Endocrinology, Cairns Hospital, Cairns, QLD 4870, Australia; Menzies School of Health Research, Charles Darwin University, Darwin, NT 0811, Australia; Department of Diabetes and Endocrinology, Cairns Hospital, Cairns, QLD 4870, Australia

**Keywords:** McCune-Albright syndrome, fibrous dysplasia, denosumab, acromegaly, chronic myeloid leukemia, imatinib, hypocalcemia

## Abstract

McCune-Albright syndrome (MAS) is a rare mosaic disorder characterized by the classic triad of fibrous dysplasia of bone (FD), café-au-lait skin macules, and hyperfunctioning endocrinopathies. MAS is caused by a postzygotic mutation in the G-protein alpha subunit (GNAS) gene resulting in G-protein α-subunit somatic activation. There is no approved treatment for MAS. We present the case of a 43-yr-old male carpenter with severe polyostotic FD and adult-onset growth hormone (GH) excess who was treated with denosumab and somatostatin analog, complicated with a diagnosis of chronic myeloid leukemia (CML). The patient had multiple skeletal lesions, resulting in pain on movement and neurovascular compromise of the left arm. A forequarter amputation was considered to treat a large clavicular lesion, however, involvement of his thoracic cage resulted in significant cardiopulmonary impairment, including restrictive lung disease, and the surgery was deemed too risky. Denosumab was commenced after failed intravenous bisphosphonate for pain management, resulting in alleviation of pain. Screening of endocrinopathy revealed GH excess with an elevated Insulin-like Growth Factor-1 (IGF-1) level and 7 mm pituitary adenoma. Lanreotide was commenced as a medical therapy, resulting in a reduction in IGF-1 levels. Over 9 mo into the denosumab treatment, the patient was diagnosed with CML in the context of routine full blood examination. The patient achieved a hematological remission with imatinib. Polyostotic FD can lead to serious complications from deformities of the skeleton, including cardiopulmonary complications. This case represents a patient with a severe spectrum of MAS/FD with a diagnosis of CML. We postulate that CML is unlikely due to the MAS, as the two have different pathogenic pathways. Denosumab is effective in pain management, however, it should be used with caution, and there are no large studies to guide long-term management. Evaluation and management of MAS should also include detailed endocrinopathy assessment and screening, even in adulthood.

## Graphical Abstract

**Graphical Abstract:**
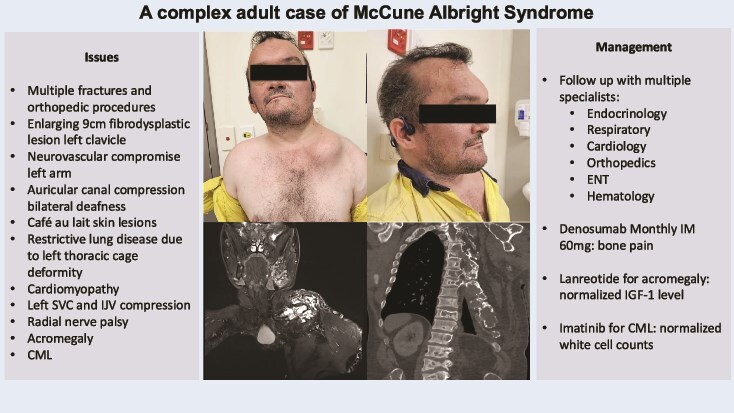

## Introduction

McCune-Albright syndrome (MAS) is a rare disorder due to somatic gain-of-function mutations of the GNAS gene, located on chromosome 20q13.3. It is recognized by the triad of polyostotic fibrous dysplasia (FD), café-au-lait macules, and hyperfunctioning endocrinopathies.^1^ The prevalence of MAS is approximately 1 in 1 00, 000 to 1 in 1, 000, 000 people.^2^^,^^3^

The resulting disease is mosaic with a broad clinical spectrum. FD may involve one (monostotic) or multiple (polyostotic) bones, and its severity can range from an incidental radiological finding to severe and disabling disease.^3^ There is no approved medical treatment to control pain and disease activity in FD. Bisphosphonates have been used in the management of patients with FD-related bone pain, however, those with a high skeletal burden may not respond to treatment with these agents.^4^ Denosumab has been shown to be effective, without serious adverse events, in those with FD refractory or intolerant to bisphosphonate therapy.^4^^,^^5^

Chronic myeloid leukemia (CML) is one of the most common leukemias that occur at any adult age, but typically in adults over the age of 40. Hematological disorders are rare in FD/MAS, although two pediatric cases of bone marrow failure due to hyperthyroidism and loss of hematopoietic marrow space have been reported.^6^

We report a case of denosumab use in a male adult patient with severe polyostotic FD who had a subsequent diagnosis of acromegaly, thus meeting the criteria for MAS. He also then developed CML. His disease profile is an example of the multiple life-threatening complications that can occur in MAS.

## Case description

A 43-yr-old male carpenter was referred to the Adult Endocrinology Department in 2019 for management of polyostotic FD. He migrated to Australia 7 yr earlier and had a medical history of polyostotic FD since childhood, which had been managed in his home country. Pituitary screening performed until the age of 18 showed no evidence of hyperfunctioning endocrinopathies. Multiple skeletal sites were involved, including his lower limbs, left clavicle, left thoracic cage, proximal humerus, auricular canals, and craniofacial bones. These resulted in complications of a severe ventilatory defect from the left thoracic cage deformity, dilated cardiomyopathy with an ejection fraction of 46%, and compression of the left superior vena cava and the internal jugular vein (IJV). His main symptoms were dyspnea on exertion and severe pain (Visual Analog Scale [VAS]: 7 out of 10) around the left clavicle, exacerbated by the physically demanding nature of his carpentry work. The auricular canal compression resulted in bilateral conductive hearing impairment.

**Figure 1:**
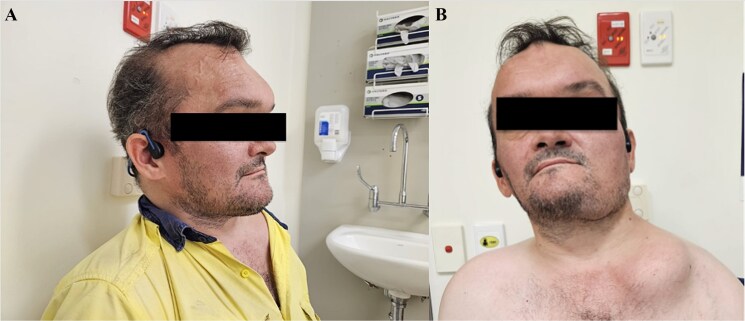
Patient photographs. (A) Facial asymmetry and craniofacial involvement. (B) Clinical photograph showing a large bony lesion in the left clavicular region and craniofacial involvement.

**Figure 2:**
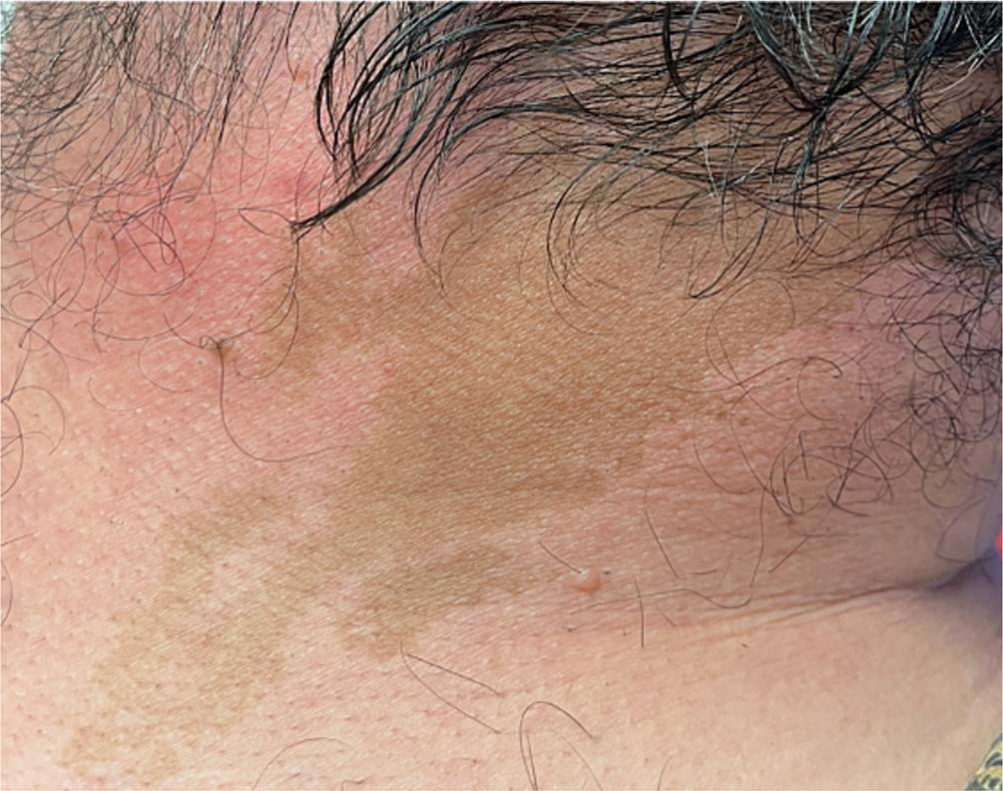
Café-au-lait spots, located on his neck.

Pertinent examination findings included facial asymmetry (Figure 1A), asymmetry of the chest wall, a large rounded bony lesion on the left clavicle (Figure 1B), and café-au-lait spots on his buttocks and neck (Figure 2). There was no thyroid goiter.

**Figure 3:**
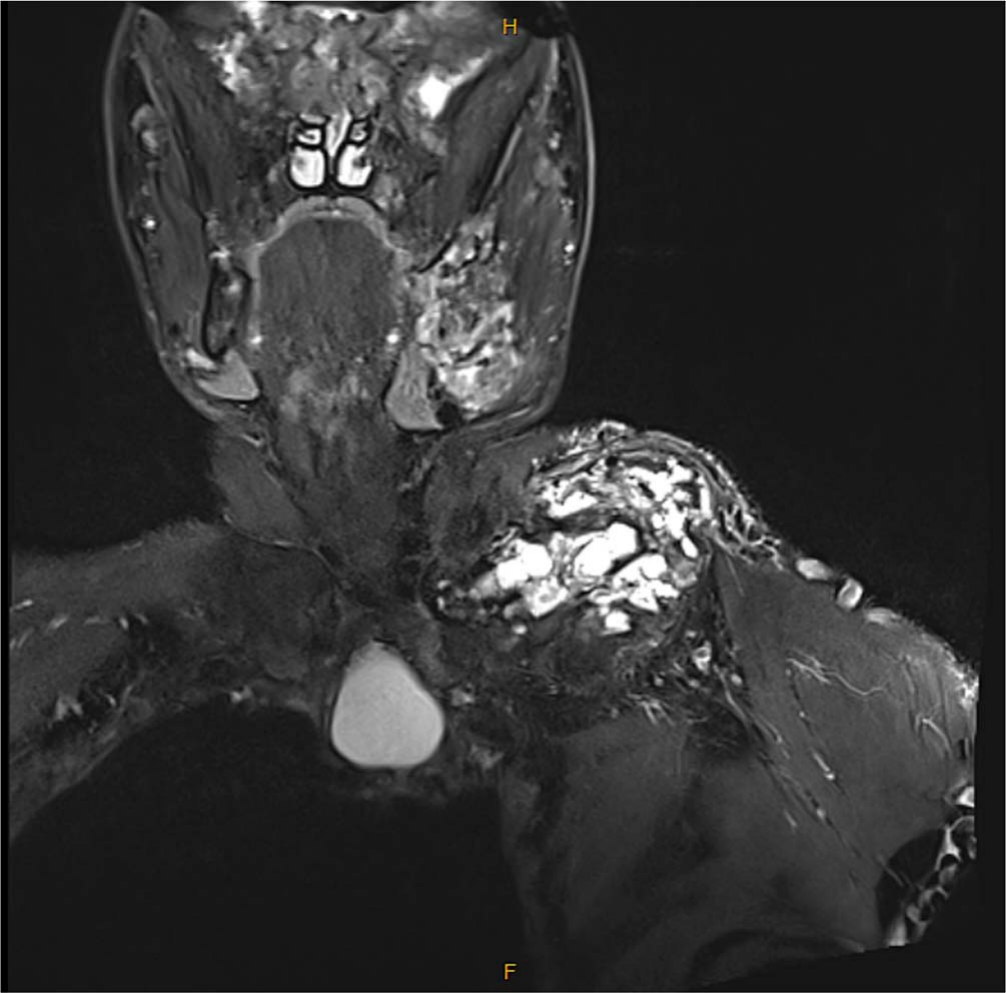
Coronal view of MRI left shoulder and clavicle with large expansile bony lesion on the left clavicle.

**Figure 4:**
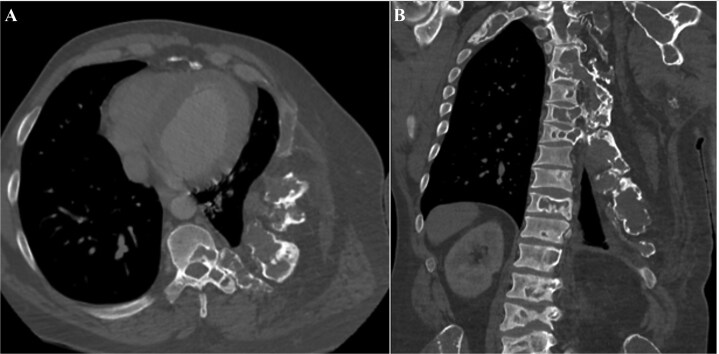
CT of the chest. (A) Transverse view of CT of the chest. (B) Coronal view of CT of the chest showing left-hemi thorax grossly reduced in volume with the expansile fibrous lesions involving multiple ribs, the left shoulder girdle, and the left half of the dorsal vertebra.

His fasting metabolic bone studies at the initial endocrinology assessment demonstrated alkaline phosphatase of 466 U/L (30-11 U/L), and elevated bone turnover markers (BTM) (CTX 2590 ng/L [100-600 ng/L]) (Table 1). There was no evidence of renal phosphate wasting. A biopsy of the left clavicle lesion showed no malignant cells on histopathology on two occasions 3 yr apart but did show fibrous tissue. An MRI of his left shoulder demonstrated a marked expansile mass, which had a maximum diameter of 9.3 cm (Figure 3). CT of his chest showed reduced size of the left hemithorax, and CT venogram confirmed vascular compression of the left IJV (Figure 4). His pulmonary function test showed a mixed obstructive and restrictive ventilatory defect, with a forced expiratory volume in one second of 1.24 L (30.4%) and a forced vital capacity of 37.8%.

**Table 1:** Laboratory test results trended over time.

***Fasting bloods***	**Baseline**	**Trough level monthly denosumab**	**Trough level 3-monthly denosumab**	**Reference range**
**Serum corrected calcium (mmol/L)**	2.39	2.17	2.65	2.10-2.60 mmol/L
**Serum phosphate (mmol/L)**	0.92	0.56	1.01	0.70-1.50 mmol/L
**eGFR (mL/min)**	>90	>90	>90	>60 mL/min
**Alkaline phosphatase (U/L)**	466	151	255	30-110 U/L
**25-OH vitamin D (nmol/L)**	59	75	55	>50 nmol/L
**Parathyroid hormone (pmol/L)**	7.1	-	2.3	1.9-8.5 pmol/L
**C-terminal telopeptide of type 1 collagen (ng/L)**	2590	420	380	100-600 ng/L
**Total P1NP (**μ**g/L)**	1648	788	586	15-80 μg/L
**IGF-1 (nmol/L)**	57	35	29 After 60 mg of lanreotide	12-34 nmol/L
**White cell count**	5.9	6.5	52.6 Diagnosis of CML confirmed on bone marrow biopsy	3.5-10.0 10^9^/L

Abbreviation: CML, chronic myeloid leukemia.

**Figure 5:**
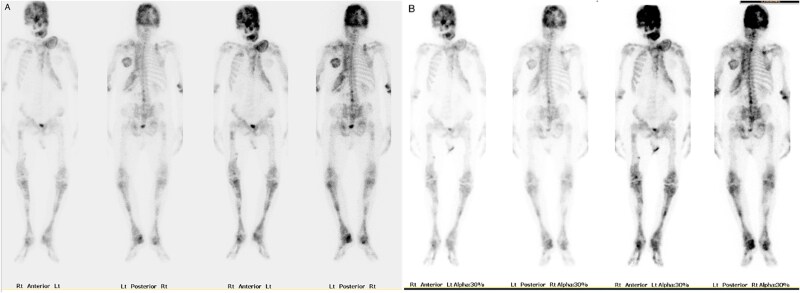
Whole-body technetium-99 m hydroxy diphosphonate bone scan. (A) Baseline, prior to zoledronic infusion. (B) Repeat scan, 6 mo post zoledronic infusion.

His initial bone scan showed increased osteoblastic activity throughout the entire skeleton, apart from the right hemithorax (Figure 5, Panel A). A trial of zoledronic acid infusion for his pain management exacerbated his pain and there was minimal change to osteoblastic activity on a repeat bone scan done 6 months after the infusion (Figure 5, Panel B).

During the assessment, a full anterior pituitary hormone panel was performed, which revealed a raised IGF-1 level of 56 nmol/L (12-34 nmol/L). The remainder of his pituitary profile was normal. An oral glucose tolerance test failed to suppress growth hormone (GH) to less than 1 μg/L. MRI of the pituitary demonstrated a 7 mm non-enhancing right hemi-pituitary lesion. Consensus from a pituitary multidisciplinary meeting was for medical management with somatostatin analog, as the patient had a high anesthetic and neuro-surgical risk given his craniofacial FD. Lanreotide was commenced, which resulted in lowering his IGF-1 level from 57 to 29 nmol/L within 3 mo.

His case was also discussed at a multidisciplinary orthopedic and cardiothoracic meeting for consideration of total claviclectomy, due to the extent of his pain and neurovascular compromise to the left arm. He was flown to a specialist orthopedic center for this operation, however, once he was reviewed in person, it was canceled because of the exceedingly high risks of surgery, including major vascular damage and potential bleeding into the thorax. The consensus was that if the clavicular lesion continued to expand, a forequarter amputation would be necessary.

Monthly 60 mg denosumab was therefore commenced for pain management, supplied with special approval from the local hospital pharmacy. The patient’s pain improved with mild flares after exercise (VAS: 4/10). His BTM levels significantly reduced (CTx: 420 ng/L [100-600 ng/L], P1NP 788 [5-80 μg/L]) 1 mo after the first dose of denosumab. There were no side effects from denosumab other than asymptomatic mild hypocalcemia. After 6 mo of monthly denosumab, the dosing regime was stretched to 3 monthly, with recurrence of pain and mild rebound hypercalcemia toward the end of each 3-mo period.

During routine follow-up, the patient’s full blood count showed a raised white cell count suspicious for underlying hematological malignancy (Table 1). Urgent hematology referral and a bone marrow biopsy were performed. The patient was diagnosed with CML and was commenced on BCR-ABL tyrosine kinase inhibitor (TKI) (imatinib). He has achieved hematological remission with imatinib without any major adverse events.

This patient continues to work as a carpenter, full-time, despite requiring intense follow-up with multiple specialists for his MAS-related complications: endocrinology, respiratory, cardiology, orthopedics, ENT, and more recently hematology.

## Discussion

This case describes an adult patient with severe FD/MAS that resulted in extensive skeletal involvement with acromegaly. Management required multidisciplinary input and medical treatments, including denosumab for his FD, lanreotide for his acromegaly, and imatinib for the recent diagnosis of CML. To our knowledge, this is the first reported case of CML diagnosis in a patient with MAS.

Polyostotic FB is a hallmark of MAS. It presents at a younger age and affects predominantly the craniofacial bones, thoracic cages and long bones. Management options for FD have been mainly surgical intervention, targeting stabilization to prevent impending fractures and correcting deformities. Restrictive lung disease and cardiac complications from involvement of the thoracic cage are rare complications of FD.^7^^,^^8^ In some cases, supplemental oxygen has been used. Despite the cardiac and ventilatory impairment described in our case, our patient had only a mild reduction in daily functional capacity at this stage, however, his anesthetic risks were significant.

Denosumab has been advocated as a potential treatment for FD/MAS, as RANK-ligand expression is upregulated in skeletal lesions in patients with FD, which correlates with the disease burden.^9^ Several observational studies have reported on the successful use of denosumab therapy in patients with FD/MAS, particularly in bisphosphonate refractory patients.^5^^,^^7^^,^^8^ In particular, the observational study conducted by Trojani et al. showed an average reduction of 4.9 points in the VAS score within their cohort.^8^ In addition to improvements in pain, denosumab may provide additional benefits of reducing bone lesion activity and volume and improving other symptoms associated with FD/MAS complications. De Castro et al. showed marked reductions in the BTMs, reduced bone lesion activity, and improved pulmonary function in a participant with thoracic FD involvement.^7^

The optimal dose and frequency of denosumab remain unclear. A case series on the use of denosumab in patients with FD showed that the standard denosumab regimen as per osteoporosis management was insufficient to maintain the reduced BTM in patients with FD, whereas a 3-monthly regimen effectively decreased BTM levels.^4^ High-dose denosumab (120 mg) has also been used without serious adverse events, highlighting that the low-dose denosumab is unlikely to improve pain and effective BTM reduction.^11^ In our case, the decision was made to give 60 mg monthly injections, with the view to extending the interval based on clinical and biochemical progression. His pain responded very well to treatment.

There is no available data on the optimal total duration of denosumab and further management once patients are in clinical and biochemical remission. The risk of osteonecrosis of the jaw with antiresorptive agents is well emphasized in the literature,^9^ however, no cases have been reported in relation to antiresorptive therapy in patients with FD/MAS. The primary concern regarding denosumab discontinuation is rebound hypercalcemia and elevated BTM, which have been reported in two pediatric cases.^10^ In adult populations, no symptomatic hypercalcemia was seen after withdrawal from denosumab during a 3-yr follow-up period. Only one patient had a mild hypercalcemia of 2.73 mmol/L, 5 mo after the last dose of denosumab.^5^ A pilot study^7^ showed one participant with severe hypercalcemia secondary to vomiting and two participants with mild asymptomatic hypercalcemia. All of the participants received zoledronic acid infusion a month after the final dose of denosumab. Our patient has had no significant side effects from denosumab apart from mild asymptomatic variations in calcium. Administration of intravenous zoledronic acid will be re-considered given our patient’s significant skeletal disease burden. It is unclear whether he will be able to cease denosumab in the future.

This complex case was complicated further by a diagnosis of CML. Hematological malignancies are not associated with MAS, although a case of bone marrow failure was reported in the literature.^6^ There were no reports of hematological malignancy from the Dutch National Pathology Registry data of patients with MAS/FD.^13^ A systemic review of the incidence of malignancy in patients with acromegaly,^11^ reported an increased risk of hematological cancers, primarily malignant lymphoma and leukemia, with a standardized incidence rate of 1.89. CML is a myeloproliferative neoplasm characterized by the fusion of the genes on chromosome 9 and chromosome 22, resulting in the BCR-ABL1 gene activating the tyrosine kinase enzyme. Given this pathogenesis, we postulate that CML is an incidental diagnosis rather than related to FD/MAS pathogenesis, as MAS involves the increased activation of the cyclic AMP-protein kinase signal transduction pathway, from the somatic gain of mutation in the GNAS gene on chromosome 20q13-13.29.^3^ He may, however, have had an increased risk of hematological malignancy due to GH excess.

TKIs including oral imatinib, are the first line therapy in CML given their action specifically on the tyrosine kinase receptor. There have been no reports of denosumab associated with CML, however, hypocalcemia has been reported with TKIs, including imatinib.^12^ A review on TKIs associated with electrolyte disorders^13^ demonstrated that TKIs can cause hypocalcemia and hypophosphatemia due to their act on the platelet-derived growth factor receptor on osteoclasts and osteoblasts, in turn decreasing bone turnover and osteoclastogenesis. The combination of denosumab and imatinib therefore has a risk of hypocalcemia and regular monitoring of electrolytes is recommended.^12^^,^^13^

In this case, the patient was diagnosed with acromegaly in adulthood during a routine assessment, despite negative test results in his childhood. As endocrinopathies are a feature of MAS/FD, repeated detailed endocrine evaluations including pituitary assessments are essential.^3^

Acromegaly, excess GH caused by a pituitary tumor, is observed in 10% to 20% of MAS patients.^14^ The diagnosis of acromegaly is generally made during adulthood. In a review of all reported acromegaly cases in patients with MAS,^14^ the average age at diagnosis of acromegaly in patients with MAS, including the pediatric population was 24.4 and 30.1 yr in those patients diagnosed during adulthood. Hyperprolactinemia was present in 81% of cases. Craniofacial FD involving the skull base is almost always seen in MAS patients with GH excess. This can cause the acromegaly diagnosis to be challenging, as the dysmorphic craniofacial features could be masked.^3^^,^^14^^,^^15^ Exposure to excess GH likely worsens craniofacial FD, as GH and IGF-1 levels correlate with worsening craniofacial FD features.^16^ This highlights the importance of regular endocrinopathy screening in those patients with FD/MAS, even post-transitioning to adulthood.^14^

Pituitary surgery in MAS is particularly challenging due to the craniofacial bone involvement of FD, a high recurrence rate, increased bleeding risk, and poor healing.^1^^,^^17^ It is also unlikely to normalize GH/IGF-1 excess, as evident by the review of reported cases.^14^ Therefore, the 2019 best practice management guidelines for FD/MAS suggest medical therapy with a somatostatin analog, either alone or in combination with pegvisomant. Radiotherapy is not recommended due to the risk of malignant transformation in craniofacial bones. In our case, it is unclear whether some of his comorbidities were a result of acromegaly, such as his dilated cardiomyopathy and deafness, and whether these will improve with a somatostatin analog.

## Summary

In summary, our case highlights the challenging clinical spectrum of patients presenting with MAS/FD and the importance of systematic endocrinopathy evaluation. It is also the first case reporting CML in a patient with MAS/FD and acromegaly. Close follow-up is necessary in this case due to the addition of imatinib for CML. We demonstrate that the use of denosumab and somatostatin analogs in this case so far has been well tolerated. This case highlights the importance of multidisciplinary input and the need for careful long-term follow-up of patients with MAS.

## Data Availability

Data available on request.
